# Animal infection studies of two recently discovered African bat paramyxoviruses, Achimota 1 and Achimota 2

**DOI:** 10.1038/s41598-018-31193-z

**Published:** 2018-08-24

**Authors:** Jennifer Barr, Shawn Todd, Gary Crameri, Adam Foord, Glenn Marsh, Leah Frazer, Jean Payne, Jenni Harper, Kate S. Baker, Andrew A. Cunningham, James L. N. Wood, Deborah Middleton, Lin-Fa Wang

**Affiliations:** 1 CSIRO Australian Animal Health Laboratory, Geelong, Australia; 2 Programme in Emerging Infectious Diseases, Duke-NUS Medical School, Singapore, 169857 Singapore; 3 Institute of Zoology, Zoological Society of London, London, NW1 4RY United Kingdom; 4 Department of Veterinary Medicine, University of Cambridge, Cambridge, CB3 0ES United Kingdom; 5 Institute for Integrative Biology, University of Liverpool, L69 7ZB Liverpool, United Kingdom

## Abstract

Bats are implicated as the natural reservoirs for several highly pathogenic viruses that can infect other animal species, including man. Here, we investigate the potential for two recently discovered bat rubulaviruses, Achimota virus 1 (AchPV1) and Achimota virus 2 (AchPV2), isolated from urine collected under urban bat (*Eidolon helvum*) roosts in Ghana, West Africa, to infect small laboratory animals. AchPV1 and AchPV2 are classified in the family *Paramyxoviridae* and cluster with other bat derived zoonotic rubulaviruses (i.e. Sosuga, Menangle and Tioman viruses). To assess the susceptibility of AchPV1 and AchPV2 in animals, infection studies were conducted in ferrets, guinea pigs and mice. Seroconversion, immunohistological evidence of infection, and viral shedding were identified in ferrets and guinea pigs, but not in mice. Infection was associated with respiratory disease in ferrets. Viral genome was detected in a range of tissues from ferrets and guinea pigs, however virus isolation was only achieved from ferret tissues. The results from this study indicate Achimota viruses (AchPVs) are able to cross the species barrier. Consequently, vigilance for infection with and disease caused by these viruses in people and domesticated animals is warranted in sub-Saharan Africa and the Arabian Peninsula where the reservoir hosts are present.

## Introduction

New and emerging viral infections impose a significant burden on human health and on the world economy. The majority of emerging infectious diseases affecting humans today are of animal origin with approximately three quarters arising from wildlife^1^. Bats have been shown to harbour more zoonotic viruses than other mammalian species^2,3^ and are implicated in outbreaks of a number of highly pathogenic zoonotic viruses, including filoviruses, coronaviruses, paramyxoviruses and reoviruses. Ebola virus (EboV) RNA and antibodies have been discovered in African fruit bats^4^. The largest human outbreak of EboV occurred in West Africa in 2013–2014, resulting in nearly 30,000 infections and 11,000 deaths and took over a year to contain^5^. SARS coronavirus emerged in China in 2002 and infected over 8000 people causing 774 deaths^6^ and the MERS coronavirus continues to infect people and cause death in the Middle East^7^. Bats have since been found to harbour a multitude of coronaviruses closely related to SARS and MERS^8,9^. Pteropid bats are the reservoir hosts for the deadly henipaviruses, Nipah virus (NiV) and Hendra virus (HeV)^10,11^. NiV continues to cause fatal encephalitis in humans almost annually in Bangladesh, while HeV has spilled-over into horses in Australia nearly every year since 2004 and has killed four people^12,13^. Orthoreoviruses have been isolated from bats and humans in Southeast Asia where they have caused flu-like illness in people^14^. Known zoonotic viruses of bat origin continue to be of concern for human and animal health, and active surveillance provides our best option for monitoring these agents as well as identifying novel pathogens of zoonotic potential.

The recently discovered rubulaviruses, Achimota virus 1 and Achimota virus 2 (AchPV1 and AchPV2), were isolated from bat (*Eidolon helvum)* urine samples collected beneath urban bat roosts in Ghana, West Africa^15^. AchPV1 and AchPV2 are newly recognised viral species in the family *Paramyxoviridae*, where they cluster with other bat rubulaviruses. Despite being discovered in the same study, the AchPVs are not nearest-phylogenetic relatives and share only 31 to 64% protein amino acid identities^15^. Their relationship to each other is similar to their relationships with other bat rubulaviruses such as Sosuga (SosPV), Menangle (MenPV) and Tioman (TioPV) viruses (sharing 58–70% N protein amino acid sequence identities), which have been shown to cause human infection. SosPV was isolated from a wildlife biologist studying bats and rodents in Africa in 2012 and is believed to be the causative agent for a severe flu-like illness and skin rash^16^. Follow up investigations revealed the presence of this virus in the spleen of *Rousettus aegyptiacus* bats^17^. MenPV first emerged in a piggery in NSW, Australia, in 1997 causing reproductive disease in pigs^18^. Two piggery workers had flu-like illness during the outbreak and were later found to have MenPV neutralising antibodies. Serological evidence of MenPV infection was also found in flying foxes roosting near the piggery and the virus was later isolated from *Pteropus alecto* urine^18,19^. TioPV was isolated from pteropid bat urine on Tioman Island in 2001 during the search for the reservoir host of Nipah virus^20^. It was later found that humans on the island had neutralising antibodies to TioPV, although no associated disease has been reported^21^.

Based on what is known of these closely related bat rubulaviruses, the potential of AchPV1 and AchPV2 to infect and cause disease in other species is worthy of further investigation. Serological surveys of *Eidolon helvum* populations in Africa have been conducted for AchPVs and have shown a widespread presence of neutralising antibodies^15^. In addition, a survey of human sera collected from Ghana and Tanzania detected AchPV2 neutralising antibodies in three of 442 samples tested, however no neutralising antibodies to AchPV1 were detected in these sera. Two of the antibody positive samples were from healthy adults and one was from a febrile paediatric patient^15^. These data suggest that AchPV2 is zoonotic, but whether AchPV1 is zoonotic remains unknown.

To further investigate the infection potential of AchPV1 and AchPV2, we conducted studies in three species of small laboratory animal; ferret (*Mustela putorius furo*), guinea pig (*Cavia porcellus*) and mouse (*Mus musculus domesticus*). First, we conducted observational studies to determine the susceptibility of these animals to infection by AchPV1 or AchPV2. Second, time course studies were performed using AchPV2 to obtain data on viral replication sites and potential routes of transmission.

## Results

### Observational study with AchPV1 and AchPV2 in ferrets

Two adult male ferrets aged 11–13 months were given 10^5^ TCID_50_ AchPV1 oronasally in 1 ml of inoculum and another two adult male ferrets aged 11–13 months were given 10^5^ TCID_50_ AchPV2 oronasally in 1 ml of inoculum. The animals were observed daily for clinical signs and then electively euthanased at 21 days post challenge (pc).

One of the two ferrets exposed to AchPV1 remained clinically well and was electively euthanased at the end of experiment on day 21 pc. The other ferret showed signs of upper respiratory tract infection (sneezing, coughing) and weight loss from day 1 pc, and was euthanased at day 14 pc when it had reached a predetermined humane endpoint of 10% bodyweight loss. Post mortem examination revealed a pleural effusion and bronchopneumonia of the right intermediate lung lobe. Each ferret developed neutralising antibody against AchPV1, with titres of 1:320 (healthy) and 1:80 (ill) (Table 1).

**Table 1:** The serum neutralisation titres against AchPV1 and AchPV2 for ferret and guinea pig serum collected 21 days pc. The serum collected from the animals pre-challenge (day 0) were all negative. Mouse sera were also tested but the data is not shown as they didn’t seroconvert.

AchPV1		AchPV2	
Animal	SNT Titre	Animal	SNT Titre
Ferret 1	1:320	Ferret 1	>1:1280
Ferret 2*	1:80	Ferret 2	1:1280
Guinea Pig 1	1:40	Guinea Pig 1	1:160
Guinea Pig 2	1:80	Guinea Pig 2	1:40
Guinea Pig 3	1:320	Guinea Pig 3	1:80
Guinea Pig 4	1:80	Guinea Pig 4	1:320

^*^This animal became ill and was euthanized at day 14 pc instead of day 21 pc.

One of two ferrets exposed to AchPV2 remained clinically well and was electively euthanased at the end of the experiment on day 21 pc. The other ferret maintained normal play activity but showed signs of upper respiratory tract infection (sneezing, purulent nasal discharge) from day 3 pc and which resolved by day 11 pc. The animal was electively euthanased at the end of the experiment on day 21 pc. Each ferret developed neutralising antibody against AchPV2, with titres >1:1280 (healthy) and 1:1280 (ill/recovered) (Table 1).

In summary, it is unclear whether the signs of respiratory tract disease in one of two ferrets given either AchPV1 or AchPV2 were attributable to infection by the challenge virus or by co-infection of an unknown pathogen. As higher neutralising antibody titres were observed in ferrets infected with AchPV2, and as there was prior serological evidence of this virus in people, AchPV2 was selected for a time-course study with ferrets.

### Observational study with AchPV1 and AchPV2 in guinea pigs

Four adult female guinea pigs were given 10^5^ TCID_50_ AchPV1 oronasally in 1 ml of inoculum and another four adult female guinea pigs were given 10^5^ TCID_50_ AchPV2 oronasally in 1 ml of inoculum. The animals were observed daily for clinical signs and then electively euthanased at 21 days pc.

All four guinea pigs exposed to AchPV1 remained clinically well and were electively euthanased on day 21 pc. Each guinea pig developed neutralising antibody against AchPV1, with titres of 1:320, 1:80, 1:80 and 1:40 (Table 1). Similarly, all four guinea pigs exposed to AchPV2 remained clinically well and were electively euthanased on day 21 pc. Each guinea pig developed neutralising antibody against AchPV2, with titres of 1:320, 1:160, 1:80 and 1:40 (Table 1).

Based on the same rationale as for ferrets, AchPV2 was selected for a time-course study with guinea pigs.

### Observational study with AchPV1 and AchPV2 in mice

Ten mice (five female BALB/c mice aged 12 weeks and five female BALB/c mice aged over 12 months) were given 10^3^ TCID_50_ AchPV1 intranasally in 50 µl of inoculum and another ten mice (five female BALB/c mice aged 12 weeks and five female BALB/c mice aged over 12 months) were given 10^3^ TCID_50_ AchPV2 intranasally in 50 µl of inoculum. The animals were observed daily for clinical signs and then electively euthanased at 21 days pc.

All ten mice exposed to AchPV1 remained clinically well and were electively euthanased on day 21 pc. Neutralising antibody against AchPV1 was not detected in any mouse. Likewise, all ten mice exposed to AchPV2 remained clinically well and were electively euthanased on day 21 pc. Neutralising antibody against AchPV2 was not detected in any mouse. As mice had no detectable signs of disease and did not seroconvert to either AchPV1 or AchPV2, no further studies were conducted with mice.

### Time course study with AchPV2 in ferrets

#### Clinical and Pathological findings

For this study, eight adult female ferrets were given 10^5^ TCID_50_ AchPV2 oronasally in 1 ml of inoculum and then two animals were pre-allocated for euthanasia on each of days 6, 8, 10 and 21 pc. All eight ferrets in this study showed a mild but significant increase in rectal temperature over baseline on day 4 pc (p = 0.02), and a mild but significant loss of bodyweight compared to baseline on days 4 (p = 0.03) and 5 (p = 0.0004) pc (Supplementary Tables S1 and S2). Otherwise, the animals remained clinically well until elective euthanasia, apart from one of two ferrets scheduled for euthanasia on day 8 pc. This ferret (#9) showed signs of upper respiratory tract infection (sneezing, serous and then purulent nasal discharge) between days 2 and 6 pc and was euthanased on humane grounds on day 6 pc following markedly decreased play activity. Other than ferret #9, no significant gross abnormalities were observed at post mortem examination in any of the ferrets.

The three ferrets euthanased on day 6 pc had minor histopathological changes associated with viral replication. Ferret #12 (day 6 pc) showed very mild acute bronchiolitis. Immunohistochemistry revealed viral antigen in germinal centres of the retropharyngeal lymph node but not in other tissues. In ferret #14 (day 6 pc), significant histopathological changes were confined to mild acute tonsillitis, and viral antigen was detected in the tonsillar and pharyngeal epithelium, retropharyngeal lymph node (particularly the parafollicular areas), bronchial epithelial cells, bronchus-associated lymphoid tissues (BALT), perivascular spindle cells in lung, and germinal centres and periarteriolar lymphoid sheaths of the spleen. In ferret #9, euthanased on day 6 pc with respiratory disease, there was moderately severe acute bronchiolitis, hyperplasia of the BALT, excess mucus production by bronchial glands, and focal lipoid pneumonia consistent with chronic bronchial disease. Post mortem examination of ferret #9 also revealed marked nodular hyperplasia of the liver with hepatic steatosis, but this lesion was considered to be unrelated to virus exposure as no AchPV2 viral antigen was detected in the liver.

**Figure 1:**
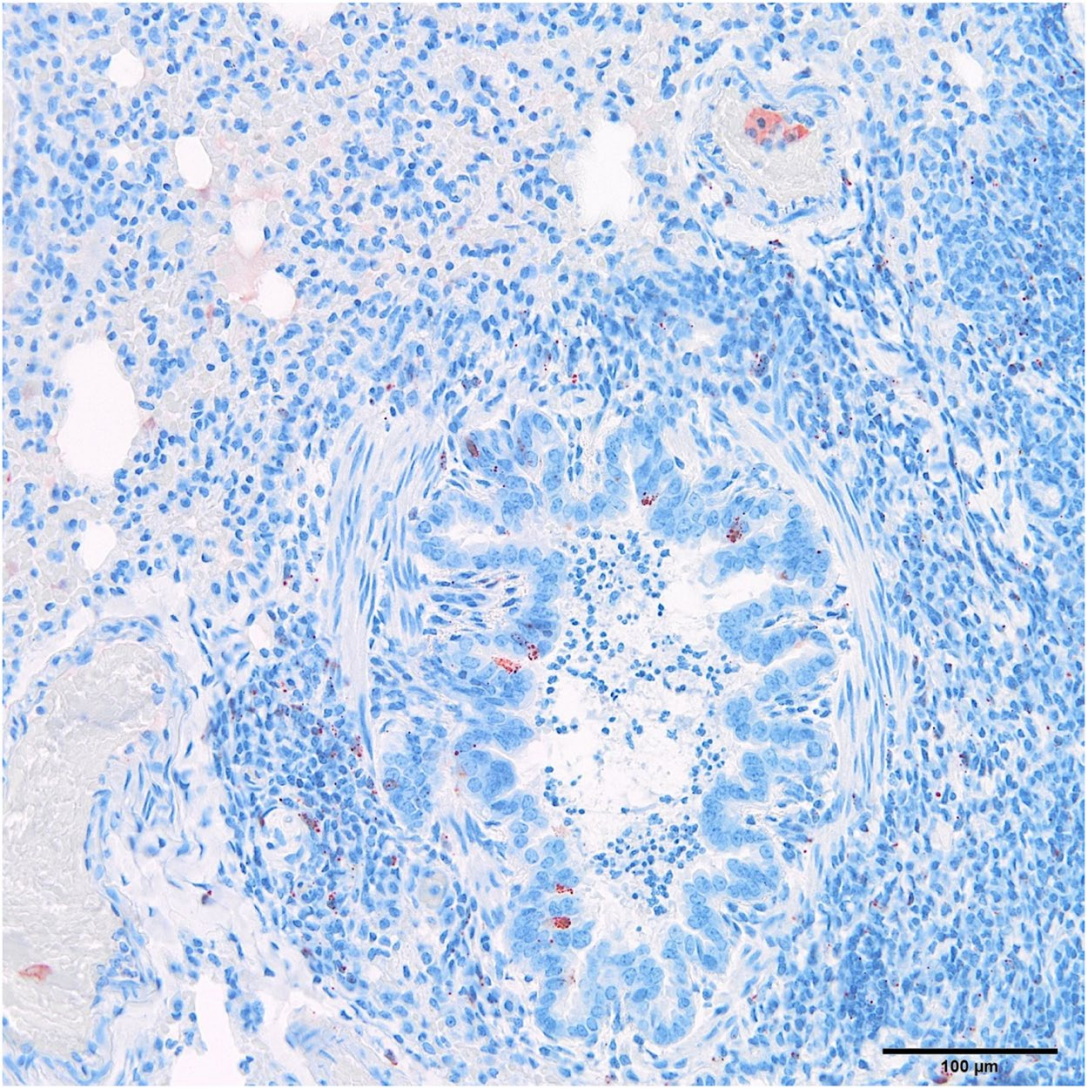
Viral antigen in bronchiolar epithelial cells and BALT in ferret #9 (polyclonal rabbit anti-AchPV2): note also intraluminal acute inflammatory infiltrate, of uncertain pathogenic significance.

In ferret #9, AchPV2 viral antigen was identified in tonsillar and pharyngeal epithelium, germinal centres, parafollicular area and medulla of the retropharyngeal lymph node, tracheal epithelium, bronchial and bronchiolar epithelium (Fig. 1), BALT and perivascular connective tissues of the lung, bronchial and mediastinal lymph node, periarteriolar lymphoid sheaths and red pulp of the spleen, mononuclear cells in the intestinal lamina propria and cells either within or lining the hepatic sinusoids.

In the single ferret euthanased on day 8 pc (ferret #16), there was mild focal acute tracheitis and bronchiolitis. The distribution of viral antigen was similar to ferret #9, with the addition of occasional bile duct epithelial cells and mononuclear cells of the portal triads, gut associated lymphoid tissue (GALT), and transitional epithelial cells in the bladder.

The two ferrets euthanased on day 10 pc showed only small amounts of viral antigen by immunohistochemistry. Ferret #11 (day 10 pc) had mild focal acute bronchiolitis, and detection of viral antigen was limited to small amounts in tonsillar lymphoid tissue, bronchiolar epithelial cells, periarteriolar lymphoid sheaths of the spleen, and a diffuse scattering throughout the retropharyngeal lymph node. Ferret #13 (day 10 pc) also showed very mild acute bronchiolitis, with AchPV2 viral antigen confined to scattered gastric epithelial cells, GALT, and sparse deposits throughout tonsillar lymphoid tissue, bronchial and retropharyngeal lymph nodes.

Of the two ferrets euthanased on day 21 pc, one (ferret #15) had very mild acute bronchiolitis and tracheitis and one had no detectable lesions. AchPV2 viral antigen was not detected in any tissue from either of these two ferrets.

#### Detection of viral genomes

AchPV2 RNA was detected by RT-qPCR in the oral swabs of 4 ferrets on day 2 pc and in all ferrets by day 4 pc until euthanasia: the highest levels were typically recorded on day 6 or 8 pc (Table 2). Similar results were seen for the nasal washes: viral RNA was detected in 3 ferrets on day 2 pc, and in all ferrets by day 4 pc until euthanasia, the highest levels typically were recorded on day 6 or 8 pc. Rectal swabs first detected AchPV2 on day 4 pc, when all ferrets were positive, the highest levels occurred on day 6 or 8 pc. Viral RNA was commonly detected in blood samples from days 2 to 21 pc. Where viral RNA was found in successive blood samples from individual animals, highest levels were recorded on day 6 or 8 pc (Table 2).

**Table 2:** Analysis of viral shedding and viraemia in ferrets by RNA detection and virus isolation. Average cycle threshold (Ct) values were obtained from testing oral and rectal swabs, nasal washes and blood from AchPV2 ferrets using RT-qPCR.

	Sample	Days Post Challenge						
		0	2	4	6	8	10	21
Ferret 9	Oral Swab	—	—	32.9	24.4***			
	Rectal swab	—	—	34.1	24.9**			
	Nasal wash	—	36.8	33	26***			
	Blood	—	—	NA	31.5			
Ferret 12	Oral Swab	—	—	32.10	21.8			
	Rectal swab	—	—	29.50	22.6			
	Nasal wash	—	—	31.40	25.1			
	Blood	—	35.9	26.50	NA			
Ferret 14	Oral Swab	—	36.9	31.90	33			
	Rectal swab	—	—	32.20	24.8			
	Nasal wash	—	—	31.40	27.1			
	Blood	—	37.8	29.10	28.7			
Ferret 16	Oral Swab	—	34.5	32.50	22.2*	24*		
	Rectal swab	—	—	32.00	22.6	23		
	Nasal wash	—	—	34.10	28.3	27**		
	Blood	—	—	31.30	30.1	20.9		
Ferret 13	Oral Swab	—	36	31.50	23.5	24	23.60	
	Rectal swab	—	—	29.50	22.8	23.4	23.70	
	Nasal wash	—	—	33.60	25.9	22.3	24.50	
	Blood	—	—	29.40	29.2	30.8	34.00	
Ferret 11	Oral Swab	—	—	31.9	22.9***	22	25.30	
	Rectal swab	—	—	30.9	22	19.6	25.50	
	Nasal wash	—	—	32.00	24.8	22.8	25.00	
	Blood	—	—	28.90	NA	27.3	36.10	
Ferret 15	Oral Swab	—	35	32.90	25.8	20.9	26.50	30
	Rectal swab	—	—	31.30	23.2	22.5	23.70	29
	Nasal wash	—	34.3	34.20	26.3	25	25.30	30.5
	Blood	—	—	24.70	NA	24	36.00	30
Ferret 10	Oral Swab	—	—	30.5	30.3	23.3	27.00	31.2
	Rectal swab	—	—	29	23.6	22.4	27.00	32.5
	Nasal wash	—	37	31.9	25.3**	24.6	26.30	28.5
	Blood	—	—	—	NA	NA	33.80	—

KEY:—indicates sample was negative (Av Ct > 38); NA indicates sample was unavailable for testing; ^*^indicates virus re-isolated at neat dilution; ^**^indicates virus re-isolated at 1:5 dilution and ^***^indicates virus re-isolated at 1:50 dilution.

**Figure 2:**
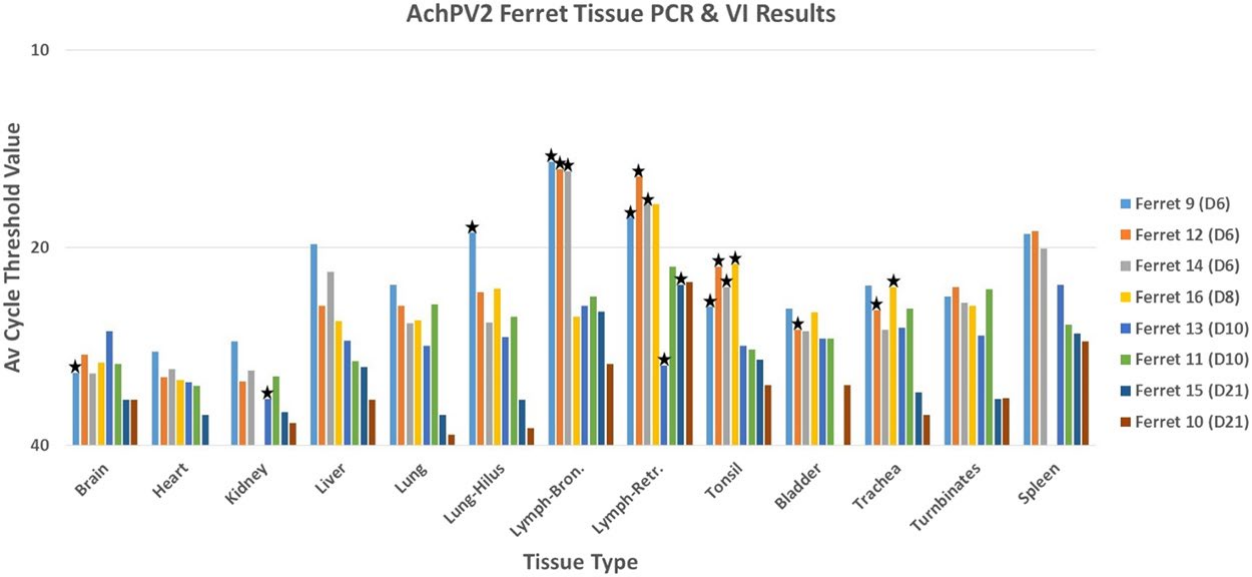
Analysis of virus infection in ferrets by RNA detection and virus isolation. Average cycle threshold (Ct) values were obtained from testing tissues from AchPV2 ferrets using RT-qPCR. Stars indicate samples that AchPV2 was re-isolated from.

All tissue samples analysed from ferrets #9, #12 and #14 euthanased on day 6 pc were positive for viral RNA (data summarised in Fig. 2), with the highest levels in bronchial and retropharyngeal lymph nodes and the lowest levels in heart, kidney and brain. Each tissue sample tested from ferret #16 (euthanased 8 days pc) was also positive for viral RNA, with the highest reading in retropharyngeal lymph node. On day 10 pc, ferrets #11 and #13 exhibited generally similar distribution and quantities of viral RNA to the animals above. However, on day 21 pc the levels of viral RNA in ferrets #10 and #15 were substantially lower, and largely limited to the retropharyngeal and bronchial lymph nodes and the spleen.

#### Virus isolation

Virus was reisolated from the nasal wash and from the oral and rectal swabs of ferret #9 on day 6 pc, the nasal wash of ferret #10 on day 6 pc, the oral swab of ferret #11 on day 6 pc, and from the oral swabs of ferret #16 on days 6 and 8 pc, plus the nasal wash of this ferret on day 8 pc (Table 2).

Virus was reisolated from ferrets killed on day 6 pc from tonsil, bronchial and retropharyngeal lymph nodes, lung and brain (ferret #9); trachea, tonsil, bronchial and retropharyngeal lymph nodes, and bladder (ferret #12); and tonsil and bronchial and retropharyngeal lymph nodes (ferret #14) (Fig. 2). Virus was reisolated from tonsil and trachea of ferret #16 (which was killed on day 8 pc) and from the retropharyngeal lymph node and kidney of ferret #13 (day 10 pc), but not from ferret #11 (day 10 pc). Virus re-isolation from ferrets killed on day 21 pc was limited to the retropharyngeal lymph node of ferret #15.

#### Serology

No neutralising antibodies were observed at day 2 or 4 pc but by day 6 low levels of neutralising antibodies were detected in the ferrets (Table 4). They showed an increase in neutralising antibody titre across the time points, with the animals bled at day 21 pc having neutralising antibody titres >1:1280.

### Time course study with AchPV2 in guinea pigs

#### Clinical and Pathological findings

For this study, eight adult female guinea pigs were given 10^5^ TCID_50_ AchPV2 oronasally in 1 ml of inoculum and then two animals were pre-allocated for euthanasia on each of days 6, 8, 10 and 21 pc. In guinea pigs, there were no significant differences in temperature or bodyweight over baseline up to day 6 pc, and the animals remained clinically healthy until elective euthanasia (Supplementary Tables S3 and S4). No significant gross abnormalities were observed at post mortem examination, apart from enlarged bronchial lymph nodes in one guinea pig euthanased on day 6 pc. The only histopathological changes observed were: mild acute tracheitis in all animals, two animals with mild acute bronchitis and/or bronchiolitis, and four with mild chronic interstitial pneumonia attributable to inhalation of plant material. In contrast to the observations in ferrets, the pattern of respiratory tract lesions did not correlate with the time post-exposure to AchPV2; very few histopathological changes were observed and all sections of tissues from all guinea pigs were negative for AchPV2 antigen by immunohistochemistry.

#### Detection of viral genomes

Low levels of AchPV2 were detected in the oral swab of one guinea pig on day 6 pc and of another on day 8 pc (Table 3). Rectal swabs were positive in four of six guinea pigs on day 8 pc, and from one guinea pig on day 10 pc. Viral RNA was found in the blood of one guinea pig on day 6 pc and of another on day 8 pc (both of which had viral RNA-positive oral swabs at these times).

**Table 3:** Analysis of viral shedding and viraemia in guinea pigs by RNA detection and virus isolation. Average cycle threshold (Ct) values were obtained from testing oral and rectal swabs and blood from AchPV2 guinea pigs using RT-qPCR. AchPV2 was unable to be re-isolated from any sample.

	Sample	Days Post Challenge						
		0	2	4	6	8	10	21
Guinea Pig 1	Oral Swab	—	—	—	—			
	Rectal swab	—	—	—	—			
	Blood	—	—	—	—			
Guinea Pig 2	Oral Swab	—	—	—	37.74			
	Rectal swab	—	—	—	—			
	Blood	—	—	—	35.25			
Guinea Pig 3	Oral Swab	—	—	—	—	36.44		
	Rectal swab	—	—	—	—	35.09		
	Blood	—	—	—	—	34.71		
Guinea Pig 4	Oral Swab	—	—	—	—	—		
	Rectal swab	—	—	—	—	37.31		
	Blood	—	—	—	—	—		
Guinea Pig 5	Oral Swab	—	—	—	—	—	—	
	Rectal swab	—	—	—	—	—	—	
	Blood	—	—	—	—	—	—	
Guinea Pig 6	Oral Swab	—	—	—	—	—	—	
	Rectal swab	—	—	—	—	—	—	
	Blood	—	—	—	—	—		
Guinea Pig 7	Oral Swab	—	—	—	—	—	—	—
	Rectal swab	—	—	—	—	35.39	34.97	—
	Blood	—	—	—	—	—	—	—
Guinea Pig 8	Oral Swab	—	—	—	—	—	—	—
	Rectal swab	—	—	—	—	33.98	—	—
	Blood	—	—	—	—	—	—	—

KEY:—indicates sample was negative (Av Ct > 38).

**Figure 3:**
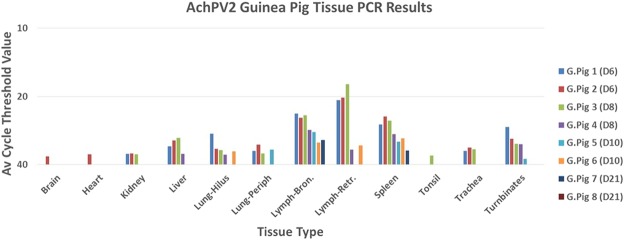
Analysis of virus infection in guinea pigs by RNA detection and virus isolation. Average cycle threshold (Ct) values were obtained from testing tissues from AchPV2 guinea pigs using RT-qPCR. AchPV2 was unable to be re-isolated from any sample.

Most tissue samples analysed from guinea pigs euthanased on day 6 and 8 pc were positive for viral RNA (data summarised in Fig. 3), with highest levels present in nasal turbinates, bronchial and retropharyngeal lymph nodes, and spleen, and lower levels in trachea, lung, and liver. Detection of viral RNA was lowest and inconsistent from heart, kidney and brain. By day 10 pc, viral genome detection was limited to bronchial and/or retropharyngeal lymph nodes, lung, spleen, and nasal turbinates (one of two animals). On day 21 pc, one guinea pig was negative by RT-qPCR for all tissues; in the other, viral genome was detected only in bronchial lymph node and spleen.

#### Virus isolation

Virus was not reisolated from any of the clinical samples, including those that were positive by AchPV2-specific RT-qPCR (Table 3). In addition, virus was not reisolated from any tissue sample, including those that were positive by AchPV2-specific RT-qPCR (Fig. 3).

#### Serology

No neutralising antibodies were observed at day 2 or 4 pc but by day 6 low levels of neutralising antibodies were detected in the guinea pigs (Table 4). There was a slight increase in neutralising antibody titre across the time points with a titre of 1:160 by day 21 pc.

**Table 4:** The serum neutralisation titres against AchPV2 for ferret and guinea pig serum collected on days 6, 8, 10 and 21 pc. Sera collected on day 2 and 4 pc were also tested but were negative for neutralising antibodies (data not shown).

Animal	SNT Titre	Animal	SNT Titre
Ferret 9* (Day6)	1:20	G.Pig 1 (Day6)	1:20
Ferret 12 (Day6)	1:40	G.pig 2 (Day6)	1:20
Ferret 14 (Day6)	1:640	G.Pig 3 (Day8)	1:20
Ferret 16 (Day8)	1:640	G.pig 4 (Day8)	1:20
Ferret 11 (Day10)	1:640	G.Pig 5 (Day10)	1:40
Ferret 13 (Day10)	>1:1280	G.pig 6 (Day10)	1:40
Ferret 10 (Day21)	>1:1280	G.Pig 7 (Day21)	1:160
Ferret 15 (Day21)	>1:1280	G.pig 8 (Day21)	1:160

^*^This animal became ill and was euthanized at day 6 pc instead of day 8 pc.

## Discussion

We investigated the potential of the two recently discovered bat rubulaviruses, AchPV1 and AchPV2, to infect laboratory animals representing three species (ferret, guinea pig and mouse). Seroconversion to both Achimota viruses in ferrets and guinea pigs indicated these animals were susceptible to infection, however mice did not seroconvert to either virus. Due to their body size, mice were given a lower dose of inoculum, and were challenged intranasally, rather than via the oronasal route used for the ferrets and guinea pigs. The difference in volume and inoculation route may account for the lack of seroconversion seen in the mice, however it is probably more likely that this species is resistant to infection. Higher levels of neutralising antibodies were observed in the ferrets and guinea pigs infected with AchPV2 compared to those infected with AchPV1. This result reflected *in vitro* data where it was observed previously that AchPV2 consistently grows to a higher titre than AchPV1 in vero and PaKi cell lines^15^. Additionally, respiratory tract disease in one of two ferrets given either AchPV1 or AchPV2 was seen in the observational studies, although it remains unclear if this was related to Achimota virus infection or was entirely due to co-infection by an unknown pathogen. AchPV2 was chosen for a time-course study based on two criteria: higher neutralising antibodies observed in ferrets and guinea pigs compared to AchPV1, and previous evidence of human infection (AchPV2 neutralising antibodies). Given more time and resources, it would be worthwhile to do an additional time course study with AchPV1, to further investigate the differences between these two viruses.

The AchPV2 time-course studies provided additional evidence that this virus can infect ferrets and guinea pigs and revealed viral replication sites and potential routes of transmission. Evidence of infection was supported by virus re-isolation from clinical specimens and post-mortem tissue samples, and viral antigen detection in tissues by quantitative real-time PCR and immunohistochemistry. Although there was evidence of mild malaise in infected ferrets (raised body temperature and weight loss), a distinct clinical syndrome with specific clinical signs was not identified in either ferrets or guinea pigs. Moreover, no histological lesions were attributed with confidence to infection by AchPV2. Mild tonsillitis, tracheitis and bronchiolitis were recorded in ferrets, but in some animals the lesions were identified without evidence of specific association with AchPV2 antigen. The ferrets were sourced from a colony free of influenza and canine distemper virus, were clinically healthy at the time of exposure to AchPV2, and were not maintained on a particulate substrate. The pathogenesis of these legions remains uncertain, although an opportunistic bacterial or other viral aetiology could not be excluded. In other tissues, such as bile duct epithelium and transitional epithelial cells of the bladder, viral antigen was seen without substantial inflammatory reaction or tissue injury.

For ferrets, following an incubation period of 5 to 6 days, AchPV2 was shed in oral and nasal secretions and the development of virus neutralising antibody was generally associated with virus clearance. The results of quantitative real-time PCR, virus re-isolation and immunohistochemistry taken together identified the major sites of AchPV2 replication in ferrets to be respiratory tract epithelium and associated lymphoid tissues. Virus was able to be re-isolated from all of the ferrets at all time points from at least one tissue or clinical sample (swab or nasal wash) but not from blood. Although virus was not re-isolated from blood, the development of viremia may be inferred by confirmation of infection within spleen and urinary tract epithelium by immunohistochemistry and viral genome detection in the blood by quantitative real-time PCR. In the absence of other clinico-pathological support for CNS infection, virus in blood may also account for the re-isolation of AchPV2 from one sample of ferret brain tissue.

Although exposure to AchPV2 resulted in production of neutralising antibodies in guinea pigs, antibody titres were much lower than for the ferrets. Significantly, patterns of viral RNA detection from guinea pigs were generally similar to those in ferrets, but virus was not re-isolated from any tissue or clinical sample from guinea pigs and viral antigen was not demonstrated in their tissues by immunohistochemistry. The sites of AchPV2 replication in guinea pigs, therefore, could not be determined with confidence. Our observations suggest that guinea pigs are less permissive to AchPV2 infection than ferrets.

When assessing the spill-over potential and working up an animal model for a novel virus, it is important to use animals from more than one species. There is no reliable method to determine the best species simply by characterising the virus, which is a big limitation of using virus discovery as a stand-alone surveillance strategy for zoonotic pathogen discovery. Therefore, it is only possible to do what is practical and feasible in terms of assessing potential spill-over hosts. Small laboratory animals such as ferrets, guinea pigs and mice, representing different mammalian orders or families, offer the most practical advantages for testing spill-over potential and, in this study, were a panel that demonstrated discriminatory power for the infection potential of the novel viruses tested.

The continued search for novel viruses in wildlife species, particularly in regions of the world where encroachment of humans and livestock into wildlife habitats is increasing, such as sub-Saharan Africa, is imperative if we are going to be able to identify disease in these regions caused by novel pathogens. New discoveries of wildlife viruses alone, however, will not inform risks to livestock or public health. Viral phylogeny and other signals of spill-over potential, such as the serosurveillance results that guided this study, are required to identify potential new health threats^22^. The Achimota viruses described in this paper demonstrate ability to cross the species barrier and may be causing undiagnosed disease in domesticated animals and humans within the wide geographical range of the bat reservoir species, *Eidolon helvum*.

## Materials and Methods

### Animals, accommodation, handling and biosafety

Ferrets were acquired from a colony free of infection by influenza H1 and H3 subtypes. Two male ferrets aged 11–13 months, four female guinea pigs, five female BALB/c mice aged 12 wks, and five female BALB/c mice aged over 12 months were used in each of the AchPV1 and AchPV2 observational studies. Eight female ferrets and eight female guinea pigs were used for the AchPV2 time course study. The animal husbandry methods and experimental design were approved by the CSIRO Australian Animal Health Laboratory’s Animal Ethics Committee (approvals AEC 1608 and AEC 1621) and were carried out in accordance with the relevant guidelines and regulations. Animals were housed at Biosafety Level 3 (BSL-3) in conventional caging systems to facilitate the expression and monitoring of natural behaviours, given complete premium dry food appropriate to the species, dietary treats, and provided with water *ad libitum*. Room temperature was maintained at 22 °C with 15 air changes per hour and humidity varied between 40 and 60%. Before any manipulation occurred, such as exposure to virus, collection of clinical samples or euthanasia, animals were immobilised with a mixture of ketamine HCl (Ketamil®: 5 mg/kg in ferrets, 16 mg/kg in guinea pigs, 75 mg/kg in mice) and medetomidine (Domitor®: 50 µg/kg in ferrets, 20 µg/kg in guinea pigs, 1 mg/kg in mice) by intramuscular or intraperitoneal (mice) injection. Where indicated, reversal was achieved with atipamazole (Antisedan®) administered by intramuscular (ferrets) or intraperitoneal (guinea pigs and mice) injection at 50% of the medetomidine volume. All animals were implanted subcutaneously with temperature-sensing microchips (Lifechip®). Staff wore powered air purifying respirators, coveralls, impervious gloves and boots while in animal rooms.

### Animal infections and sampling

For the observational studies, animals were exposed to either AchPV1 or AchPV2, previously isolated, grown and titrated in vero cells. Animal inoculation stocks were prepared as follows: after initial virus isolation, a parent stock of each virus was grown in vero cells. These parent stocks were then purified by three rounds of limiting dilution in vero cells. Finally, an animal inoculation stock was prepared from the third limiting dilution, resulting in a passage number of 6 times in vero cells from original isolation. The sequence of the animal inoculation stock was not compared to the original sequence of the isolated virus. Ferrets and guinea pigs were given 10^5^ TCID_50_ oronasally in 1 ml of inoculum (500 µl oral and 500 µl nasal), and mice were given 10^3^ TCID_50_ intranasally in 50 µl of inoculum. General clinical observations were documented daily prior to as well as post challenge (pc). Animals were weighed and their temperatures recorded daily. Animals were euthanased at either a predetermined humane endpoint or 21 days pc. Blood was collected for serology prior to virus exposure and at euthanasia. Tissues were not collected for the observational studies.

For the subsequent time course studies, ferrets and guinea pigs were exposed oronasally to 10^5^ TCID_50_ AchPV2, prepared as described above, in 1 ml of inoculum (500 µl oral and 500 µl nasal). Two animals were pre-allocated for euthanasia on each of days 6, 8, 10 and 21 pc in order to obtain preliminary data on pathogenesis and tissue tropism at likely key time-points for such an acute infection study. Nasal washes (ferrets only), oral and rectal swabs and blood samples, both in EDTA and for serum preparation, were collected from all available animals at days 2, 4, 6, 8, 10 and 21 pc. Clinical samples were collected into tubes containing PBS with antibiotic-antimycotic (Invitrogen) for virus isolation and into tubes containing MagMAX viral lysis buffer (Ambion) for RNA extraction. While under anaesthesia, rectal temperatures of ferrets were recorded by digital thermometer.

At post mortem examination of animals used for the AchPV2 time course study, the following tissues were collected for histology, immunohistochemistry, viral genome detection and virus isolation: nasal turbinates, tonsil, retropharyngeal lymph node, trachea, lung, hilar lymph node, bronchial lymph node, spleen, heart, kidney, liver, bladder and brain. Stomach, small and large intestine, pancreas, adrenal gland, ovary and uterus were also collected for histology and immunohistochemistry. Tissues were collected into tubes containing either neutral buffered 10% formalin (for histology and immunohistochemistry) or PBS plus antibiotic-antimycotic (Invitrogen) and homogenisation beads, homogenised using a bead beater, and clarified by centrifugation (for virus isolation or viral RNA detection).

### RNA extraction and Reverse Transcriptase-quantitative Polymerase Chain Reaction

For viral genome detection, RNA was extracted from tissue, blood and swab samples using the MagMAX viral RNA isolation kit (Ambion) following the manufacturers guidelines. A novel Reverse Transcriptase-quantitative Polymerase Chain Reaction (RT-qPCR), was designed that specifically targets the nucleoprotein gene (N-gene) of AchPV2. For the design process, the N-gene sequence of AchPV2 (JX051320), as well as other closely related paramyxoviruses including SosPV, MenPV and TioPV, was retrieved from GenBank. Subsequently, sequence alignments were performed using Geneious software (Version 8.1, Biomatters). Potential primer and probe regions distinctive of AchPV2 were identified from these alignments and candidate primers and probes assessed using the Primer Express 3.0.1 program (Thermofisher-Applied Biosystems). An assay spanning the 625–700 nucleotide region (75 nucleotides in length) of the N-gene of AchPV2 (JX051320), consists of forward primer: D-715 (5′-GCAGGTCTGGATCACAGTATGC-3′), reverse primer D-716 (5′-TGCCAGTCGCCTCTCATCT-3′), and probe

D-717 (5′[FAM]-TGCATGACAGCATATGATCAGCCCACT-[BHQ-1]-3′. The optimized primer and probe concentrations and assay conditions were as follows: forward primer (D-715) and reverse primer (D-716): 300 nM, probe (D-716): 200 nM. Reactions were performed using AgPath-ID One-Step RT-PCR Kit (Thermofisher-Ambion) on an AB7500 Fast instrument using the thermal cycle: 1 cycle of 45 °C 10 min, 95 °C 10 min followed by 45 cycles of 95 °C 15 sec, 60 °C 45 sec. To determine the assay efficiency, a standard curve was generated using ten-fold serial dilutions of AchPV2 RNA and was found to be 95%. The analytical specificity was investigated using a range of available paramyxoviruses, namely Newcastle disease virus, J virus, HeV, NiV and closely related rubulaviruses (MenPV and TioPV) and only the reference AchPV2 was detected by the RT-qPCR. For interpretation of results duplicate samples producing an average cycle threshold (Ct) less than 38 were considered positive.

### Virus isolation

Vero cell monolayers were grown in 96 well tissue culture plates to 80% confluency in cell media (Minimal Essential Medium containing Earle’s salts and supplemented with 2 mM glutamine, antibiotic-antimycotic and 10% fetal calf serum).

Swab media and blood were serially diluted 10 fold and 50 µl added to each well. Supernatant from centrifuged tissue homogenate was serially diluted 10 fold and 50 µl added to each well. Vero cell monolayers were observed for viral CPE seven days post infection.

### Serology

Serum was collected prior to viral challenge and again at euthanasia, and tested using a standard virus neutralisation test. Serial two-fold dilutions of test sera were prepared in duplicate in a 96-well tissue culture plate in 50 µL cell media (Minimal Essential Medium containing Earle’s salts and supplemented with 2 mM glutamine, antibiotic-antimycotic and 10% fetal calf serum). An equal volume of either AchPV1 or AchPV2 working stock containing 200 TCID_50_ was added and the virus-sera mix incubated for 30 min at 37 °C in a humidified 5% CO_2_ incubator. 100 µL of Vero cell suspension containing 2 × 10^5^ cells/mL was added and the plate incubated at 37 °C in a humidified 5% CO_2_ incubator. The plate was observed for viral CPE after seven days and the serum neutralisation titre determined as being the dilution where 100% neutralisation was observed in duplicate wells. Serum samples that showed no neutralisation of virus in duplicate wells at the starting dilution (1:10) were described as negative.

### Histology and immunohistochemistry

Formalin-fixed tissues were processed into paraffin wax and prepared into 4 µm thick sections using routine histological methods. For immunohistochemistry, antigen retrieval was performed using the DAKO PT LINK machine (Dako, Glostrup, Denmark) by heating the tissue sections to 97 °C for 30 minutes and then cooling to 70 °C in the Envision Flex Target high pH retrieval solution (DAKO) and washing for 5 minutes in Tris Buffer. After this, endogenous peroxidases were quenched by the addition of 3% H_2_O_2_ solution. Tissue sections were then incubated with the primary antibody, polyclonal rabbit antisera raised against AchPV2, at a dilution of 1:2000. The visualization system used was Envision FLEX/horseradish peroxidase (HRP) conjugated with 3-Amino-9-Ethylcarbazole (AEC) chromogen (DAKO AEC + substrate chromagen K3469). Slides were then counterstained with Lillie-Mayer haematoxylin (Australian Biostain, Traralgon, Australia) and Scotts tap water before mounting. A duplicate set of tissue sections were stained with hematoxylin and eosin stain for histological examination using routine methods.

### Statistical analysis

In the time-course study, bodyweights and rectal temperatures of ferrets, and bodyweights and microchip temperatures of guinea pigs, up to and including day 6 pc were compared using a repeated measures ANOVA followed by Dunnett’s multiple comparisons test (GraphPad Prism 7.02).

## Electronic supplementary material

Supplementary Information

## Data Availability

The datasets generated during and/or analysed during the current study are available from the corresponding author on reasonable request.
