# Supplementary material for: Animal infection studies of two recently discovered African bat paramyxoviruses, Achimota 1 and Achimota 2
Source: Sci Rep. 2018 Aug 24;8:12744. doi: 10.1038/s41598-018-31193-z (PMC6109078; doi:10.1038/s41598-018-31193-z)
Supplement: Supplementary file 1 — Supplementary Information [file 41598_2018_31193_MOESM1_ESM.docx]

Supplementary information for:

**“Animal infection studies of two recently discovered African bat paramyxoviruses, Achimota 1 and Achimota 2”**

Jennifer Barr, Shawn Todd, Gary Crameri, Adam Foord, Glenn Marsh, Leah Frazer, Jean Payne, Jenni Harper, Kate S Baker, Andrew A. Cunningham, James L. N. Wood, Deborah Middleton, Lin-Fa Wang.

Supplementary Table S1: Rectal temperatures (°C) for ferrets post challenge

|  | |  |  |  |
| --- | --- | --- | --- | --- |
| **Ferret #** | **Day 0** | **Day 2** | **Day 4** | **Day 6** |
| **9** | 39.3 | 38.7 | 39.7 | 39 |
| **10** | 38.6 | 39.8 | 39.3 | 39.6 |
| **11** | 38.8 | 39.1 | 39.5 | 39.7 |
| **12** | 39.7 | 39.8 | 40.6 | 40.3 |
| **13** | 38.5 | 40.5 | 40.2 | 40.4 |
| **14** | 39.9 | 40.7 | 40.5 | 40.1 |
| **15** | 38.9 | 39.5 | 39.1 | 39.5 |
| **16** | 39.7 | 40.9 | 40.5 | 40.1 |

Supplementary Table S2: Bodyweights (g) for ferrets post challenge

|  | |  |  |  |  |  |  |
| --- | --- | --- | --- | --- | --- | --- | --- |
| **Ferret #** | **Day 0** | **Day 1** | **Day 2** | **Day 3** | **Day 4** | **Day 5** | **Day 6** |
| **9** | 772 | 770 | 748 | 750 | 725 | 730 | 680 |
| **10** | 775 | 755 | 750 | 740 | 719 | 740 | 738 |
| **11** | 965 | 950 | 980 | 940 | 930 | 940 | 925 |
| **12** | 771 | 800 | 782 | 780 | 777 | 760 | 769 |
| **13** | 1036 | 1040 | 1042 | 1040 | 1029 | 1000 | 1006 |
| **14** | 1021 | 1015 | 1028 | 1010 | 1002 | 1000 | 1007 |
| **15** | 954 | 945 | 950 | 950 | 930 | 930 | 941 |
| **16** | 881 | 870 | 890 | 860 | 839 | 850 | 853 |

Supplementary Table S3: Microchip temperatures (°C) for guinea pigs post challenge

|  | |  |  |  |  |
| --- | --- | --- | --- | --- | --- |
| **G.Pig #** | **Day 1** | **Day 2** | **Day 4** | **Day 5** | **Day 6** |
| **1** | 38.5 | 37.7 | 38.4 | 38.1 | 39.8 |
| **2** | 39 | 38.1 | 37.9 | 37.4 | 38.1 |
| **3** | 37.6 | 38.3 | 38.3 | 38 | 38.8 |
| **4** | 38.4 | 38.8 | 38.9 | 38.2 | 38.4 |
| **5** | 38.1 | 38.9 | 38.4 | 38.1 | 38.5 |
| **6** | 39.3 | 39.2 | 39.2 | 38.9 | 39 |
| **7** | 38 | 38.6 | 38 | 38.2 | 38.4 |
| **8** | 39 | 38.8 | 38.8 | 37.7 | 38.3 |

Supplementary Table S4: Bodyweights (g) for guinea pigs post challenge

|  | |  |  |  |  |  |  |
| --- | --- | --- | --- | --- | --- | --- | --- |
| **G.Pig#** | **Day 0** | **Day 1** | **Day 2** | **Day 3** | **Day 4** | **Day 5** | **Day 6** |
| **1** | 568 | 552 | 554 | 560 | 573 | 584 | 590 |
| **2** | 557 | 586 | 588 | 585 | 588 | 596 | 602 |
| **3** | 595 | 565 | 618 | 621 | 617 | 639 | 635 |
| **4** | 687 | 656 | 669 | 670 | 676 | 687 | 685 |
| **5** | 679 | 649 | 662 | 666 | 672 | 684 | 686 |
| **6** | 681 | 664 | 672 | 683 | 662 | 672 | 668 |
| **7** | 675 | 669 | 683 | 669 | 670 | 689 | 693 |
| **8** | 708 | 673 | 677 | 687 | 681 | 685 | 682 |

Statistical analysis

In the time-course study, bodyweights and rectal temperatures of ferrets, and bodyweights and microchip temperatures of guinea pigs, up to and including day 6 pc were compared using a repeated measures ANOVA followed by Dunnett’s multiple comparisons test (GraphPad Prism 7.02).
